# Dual Logic and Cerebral Coordinates for Reciprocal Interaction in Eye Contact

**DOI:** 10.1371/journal.pone.0121791

**Published:** 2015-04-17

**Authors:** Ray F. Lee

**Affiliations:** Princeton Neuroscience Institute, Princeton University, Princeton, New Jersey, United States of America; Universiteit Gent, BELGIUM

## Abstract

In order to scientifically study the human brain’s response to face-to-face social interaction, the scientific method itself needs to be reconsidered so that both quantitative observation and symbolic reasoning can be adapted to the situation where the observer is also observed. In light of the recent development of dyadic fMRI which can directly observe dyadic brain interacting in one MRI scanner, this paper aims to establish a new form of logic, dual logic, which provides a theoretical platform for deductive reasoning in a complementary dual system with emergence mechanism. Applying the dual logic in the dfMRI experimental design and data analysis, the exogenous and endogenous dual systems in the BOLD responses can be identified; the non-reciprocal responses in the dual system can be suppressed; a cerebral coordinate for reciprocal interaction can be generated. Elucidated by dual logic deductions, the cerebral coordinate for reciprocal interaction suggests: the exogenous and endogenous systems consist of the empathy network and the mentalization network respectively; the default-mode network emerges from the resting state to activation in the endogenous system during reciprocal interaction; the cingulate plays an essential role in the emergence from the exogenous system to the endogenous system. Overall, the dual logic deductions are supported by the dfMRI experimental results and are consistent with current literature. Both the theoretical framework and experimental method set the stage to formally apply the scientific method in studying complex social interaction.

## Introduction

In order to scientifically study the human brain’s response to face-to-face social interaction, the scientific method itself may need to be improved, so that both quantitative observation and symbolic reasoning can be adapted to the situation where the observer is also observed.

Directly detecting two interacting brain responses is only now possible with the recent development of dyadic fMRI (dfMRI) [1]. Although EEG [2,3] and MEG [4] have been used for dyadic data acquisition, they are limited by their coarse spatial resolution. Also, MRI “hyperscan” (scanning a dyad from two different scanners) [5] or fMRI with recorded video for social stimulation [6] have also been utilized in the past to indirectly observe dyadic interaction. However, the video and audio links compromise some of the reciprocity between the dyad. The newly developed dfMRI has largely removed the instrumental limitations, and provides sufficient spatial and temporal resolution for directly measuring dyadic BOLD hemodynamic activation during face-to-face social interaction.

Given the fact that the observers are also observed in the dfMRI experiment, most existing syllogistic logic seems insufficient in providing a deductive reasoning framework for the analysis and synthesis of dfMRI data. To systematically address one of the essential issues in social interaction studies—the entwinement between reciprocal and non-reciprocal response [7]—a dual logic derived from abstract algebraic logic is established to provide a logical framework in which an interacting brain can be formulated by a dual system [8]. Within the dual logic framework, a propositional model was created for distinguishing reciprocal and non-reciprocal brain responses during eye contact in the dfMRI experiment. Based on this model, dual logic deductions can analytically suppress the non-reciprocity and yield the dual systems for reciprocal interaction.

Social neuroscience has accumulated a large amount of fMRI observations [9], including many empirical data on eye gazing [10]. However, in the study of social interaction [11–14], explicitly distinguishing between reciprocal and non-reciprocal components in their entwined BOLD responses has been elusive. By applying dual logic deduction in a dfMRI experiment, a data-driven dual systems [15] for reciprocal interaction during eye contact can be derived. This dual system can subserve cerebral coordinate for reciprocal interaction (CCRI), and could have broad applications in general dyadic data analysis for filtering out non-reciprocal responses. An example of CCRI used in computing reciprocal coupling modes during eye contact is provided here.

Given the vast context of the topic, the main focus of this paper is limited to dual logic, the CCRI, and an example of an application of the CCRI. The goal is to demonstrate that logical deduction can elucidate the dfMRI data and extracts deterministic aspects of the experimental results. The detailed dyadic interaction analysis is beyond the scope of this work.

## Theory

During social interaction such as eye contact, brain responses can be classified by a dual system: the exogenous system and the endogenous system. By definition, the exogenous system directly responds to any exteroceptive stimulus; the endogenous system can only be activated by interoceptive stimulus. The dual logic is proposed for deductive reasoning in these dual systems during the eye contact in the dfMRI experiment.

**Fig 1:**
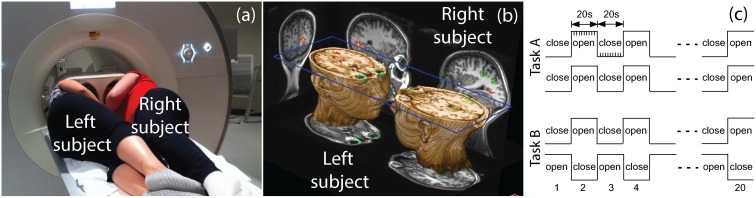
The outline of the dfMRI eye contact experiments. The (a) illustrates the dual-head coil and the dyadic placement in a commercial MRI scanner; the (b) is a 3D rendering of a dyadic anatomical data set, which illustrates the physical stimuli and BOLD responses in the experiments; the (c) depicts the temporal courses of dyadic stimuli: eye opening and/or closing in the task A and B.

In order to observe reciprocity in eye contact, the dfMRI experiment was designed as follows: Two subjects are laid on their sides, facing each other as in Fig 1a. The relative positions of their eyes and faces are locked in position as shown in Fig 1b to create a “laboratory eye contact” scenario. Two functional tasks are performed as shown in Fig 1c. During the tasks, the two subjects are verbally instructed to open and close their eyes either simultaneously in task A, or alternately in task B.

To analytically distinguish reciprocal and non-reciprocal responses in the dual system in the eye contact experiment where only binary states of task (eyes open/closed) and response (activation on/off) are of concern, the dual logic is constructed as an extension of Boolean logic. Such a construct in abstract algebraic logic is detailed in Appendix A, where the binary logic B1 and **B2** are for the exogenous and endogenous system respectively, and non-binary logic operations are defined for emergence from the exogenous system to the endogenous system.

**Fig 2:**
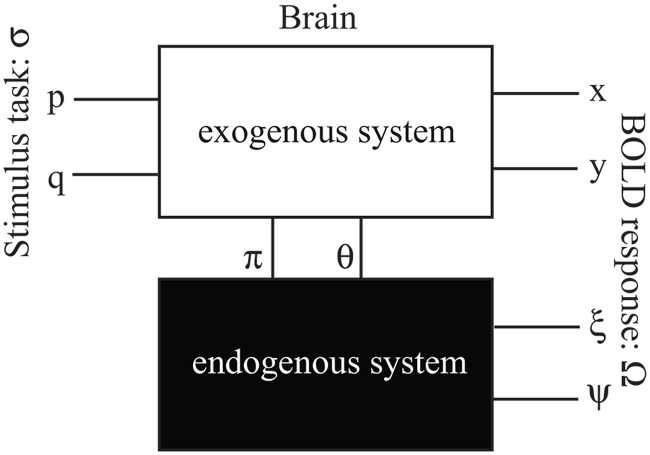
A block diagram describes the exogenous-endogenous dual systems and their stimuli-responses model. The exteroceptive stimulus σ consists of *p* and *q* states for “seeing eyes only” and “seeing face without eyes”. The arising interoceptive stimuli π and θ are the dual pairs of *p* and *q*. The BOLD response Ω consists of exogenous response Ω_+_ and endogenous response Ω_-_. The exogenous Ω_+_ can be further decomposed to two states *x* and *y* for “reciprocal responses and non-reciprocal responses; the endogenous Ω_-_ can also decomposed to two states ξ and ψ which are the dual pairs of *x* and *y*.

Within the dual logic framework, a brain in the dfMRI experiments in Fig 1 can be formulated by a stimulus-response model shown in Fig 2, in which every stimulus and response are decomposed into two states for the purpose of distinguishing reciprocity and non-reciprocity.

### Stimulus states

In the experiment shown in Fig 1, reciprocity only exists when the dyad’s eyes meet. Thus, when a subject looks at his/her partner, the exteroceptive stimulus, “I see a face”, can be decomposed into: “I see eyes only, with either a direct or averted gaze” (*p*), and “I see a face without eyes” (*q*). Here, *p* and *q* are the state variables in the logic B1 for the two dichotomous states of the exteroceptive stimulus. Their logic values are binary “1” or “0,” corresponding to “true” or “false” of the propositions *p* and *q*. Such bi-state exteroceptive stimuli can be expressed by the matrix σ for algebraic logic operation. The functionality of the σ is the disjunction of first and second row of the matrix,

$$\sigma =\;\;\left(\begin{array}{c} p\\ q \end{array}\right),f\left(\sigma \right)=\;p\vee q.$$ (1)

The *p* and *q* represent the exteroceptive stimuli of “seeing” a face. Their corresponding interoceptive stimuli π and θ can be described by the propositions “I mentalize eyes only” and “I mentalize a face without eyes” respectively [10], which represent “mentalizing face”. Notice that in this eye contact experiment scenario, “seeing” and “mentalizing” not only are two independent processes but also can coexist. To fully capture such orthogonality and to avoid any degeneracy in algebraic logic expression, the logic values of π and θ are adapted to binary “*i*” or “0”, corresponding to “true” or “false” of the variables π and θ in the logic B2. Here, “*i*” is the imaginary unit of the complex number.

### Response states

The cerebral response measured by BOLD effect (Ω) is modeled by the exogenous and endogenous dual systems. The exogenous response (Ω_+_) is activated by the exteroceptive stimulus (*p*, *q*); the endogenous response (Ω_-_) is activated by the interoceptive stimulus (π, θ). For the same reasons in describing stimulus, the response variables Ω_+_ and Ω_-_ are also depicted by logic B1 and B2 respectively. In order to untwine the reciprocal and non-reciprocal responses during eye contact, the Ω_+_ can be further decomposed into two salient states: the exogenous reciprocal state (*x*) that is only mediated by simultaneous mutual eye contact (*p*), and the exogenous non-reciprocal state (*y*) that can be induced by either *p* or *q*. Here *x* and *y* are the state variables in B1, with truth-values “1” or “0” which correspond to ON or OFF of the exogenous activations regardless of their magnitudes. The Ω_-_ can also be further decomposed into two states: the endogenous reciprocal state (ξ) and the endogenous non-reciprocal state (ψ). Here ξ and ψ are the logic variables in B2, with truth-values “*i*” or “0” which correspond to ON or OFF of the endogenous activations regardless of their magnitudes.

In the binary logic B1 and B2 sets, if both the reciprocal and non-reciprocal states share a common cerebral region within either the exogenous or endogenous system, such overlapping can be easily expressed as *x* ∨ *y* or ξ ∨ ψ. However, if overlapping occurs between the exogenous and endogenous system, Ω_+_ and Ω_-_, the truth-value may become complex 1+*i*, and quinary logic may be needed, (see Appendix A). Given that the concerns of this experiment are only binary in nature, i.e. opening/closing eyes or activation/non-activation, the B1 **- B2** logic sets seem to be mostly sufficient, except in depicting the transition between the dual systems, which is detailed in the later sections and Appendix A.

### Axioms

At the state level, to establish the logical connection between the stimulus states (*p*, *q*; π, θ) and the response states (*x*, *y*; ξ, ψ), three axioms are postulated based on self-evident truth-tables and the duality principle from De Morgan logic:

#### Axiom 1

The exteroceptive stimulus *p*, with either direct or averted gazing [16], is logically related to the disjunction of the exogenous reciprocal response (*x*) and the non-reciprocal response (*y*) by the logical connective of “material equivalence”,

p  ↔ x∨y (2)

#### Axiom 2

The exteroceptive stimulus *q* is logically related to the exogenous non-reciprocal response (*y*) in “material equivalence”,

q  ↔ y (3)

#### Axiom 3

The exogenous states (*p*, *q*; *x*, *y*) and endogenous states (π, θ; ξ, ψ) are dual pairs in the logic B1 and B2, and they obey the duality principle in De Morgan logic. Since the dual pairs for ↔ and ∨ are ⊕ (exclusive disjunction) and ∧ (conjunction) respectively, the relations between the interoceptive stimuli and their responses in endogenous system become,

$$\begin{array}{l} \pi \;\;⊕\;\;\xi \wedge \psi ,\\ \theta \;⊕\;\psi \end{array}$$ (4)

Although this axiom is a theoretical conjecture, it is supported by some computational and experimental evidence [17,18]. More importantly, the logical predictions based on these axioms are in agreement with the experimental data in this study, as well as current literature. The derivations of the three axioms are detailed in Appendix B. Based on these three axioms, all causal stimulus-response relations in this study can be logically deduced, as shown in Table 1.

**Table 1:** The dual logic.

	Exogenous system	Endogenous system
Stimulus States σ	*p* = “I see eyes, direct or averted gaze”	π = “I mentalize eyes”
	*q* = “I see face without eyes”	θ = “I mentalize face without eyes”
Response States Ω	*x* = “exogenous reciprocal social response”	ξ = “endogenous reciprocal social response”
	*y* = “exogenous non-reciprocal affective response”	ψ = “endogenous non-reciprocal affective response”
Axioms	*p* ↔ *x* ∨ *y*	π ⊕ ξ ∧ψ
	*q* ↔ *y*	θ ⊕ψ
Transformations	A: *p* ∨*q* ⇒ *x* ∨*y*	
	B: *q* ⇒ *y*	
	A-B: (*p* ∨*q*) ∧¬ *q* ⇒ *x* ∧¬ *y*	0
	B-A: *q* ∧¬ (*p* ∨*q*) ⇒ 0	B-A: π ∧θ ⇒¬ ξ ∧ ψ ∧¬ψ

### The stimulus and response states in the original tasks

To describe the stimulus in task A, substituting its two temporal stages (see and not-see) illustrated in Fig 1c into its dichotomous states *p* and *q* in Eq (1), the stimulus matrices for both left and right subjects are:

$$\sigma_{A}\left(L\right)=\;\left(\begin{array}{cc} 0 & 1\\ 0 & 1 \end{array}\right),\;\sigma_{A}\left(R\right)=\;\left(\begin{array}{cc} 0 & 1\\ 0 & 1 \end{array}\right)$$ (5)

For the task A’s cerebral response Ω_A_, although its endogenous states are elusive, its exogenous reciprocal states (*x*) are likely entwined with its exogenous non-reciprocal states (*y*). The symbolic expressions for such entwinements in the exogenous system are explicitly described in Table 2, where the response (Ω_A_ = 1) can be the results of either (*x* = 0, *y* = 1), or (*x* = 1, *y* = 0), or (*x* = 1, *y* = 1). To suppress the non-reciprocal state *y* in the Ω_A_, an additional task B is introduced, whose stimulus matrices for both left and right subject can also be derived from Fig 1c and Eq (1) as:

$$\sigma_{B}\left(L\right)=\;\left(\begin{array}{cc} 0 & 0\\ 0 & 1 \end{array}\right),\;\;\sigma_{B}\left(R\right)=\;\left(\begin{array}{cc} 0 & 0\\ 1 & 0 \end{array}\right)$$ (6)

Due to the lack of eye contact, the cerebral response in task B (Ω_B_ = 1) is the result of the only non-reciprocal response (*x* = 0, *y* = 1), as shown in Table 2. Although neither task A nor B can result in an explicit reciprocal response, the collation (defined in Appendix A) between the states in task A and B can yield the desired states. Note that the functionality *f*(σ) defined in Eq (1) for the four stimulus matrices in the Eq (5) and Eq (6) are the unit regressors in the task A and B (Fig 1c).

**Table 2:** Truth table for BOLD responses in task A, B, A-B, and B-A.

	State		Ω_A_	Ω_B_	Ω_+_	Ω_-_
Exogenous system	*x*	*y*	*x* ∨ *y*	*y*	*x* ∧ ¬ *y*	0
	0	0	0	0	0	0
	0	1	1	1	0	0
	1	0	1	N/A	1	0
	1	1	1	N/A	0	0
Endogenous system	ξ	ψ			0	¬ (ξ ∧ ψ) ∧¬ψ
	0	0			0	*i*
	0	*i*			0	*0*
	*i*	0			0	*i*
	*i*	*i*			0	0

### The composite stimuli for reciprocal interaction

Because only the proposition “I see eyes only” can induce reciprocity, the goal would be to generate a stimulus matrix that contains *p* only. Applying collation operation to the left subject, between σ_A_(L) in Eq (5) and σ_B_(L) in Eq (6), will generate two composite stimuli:

$$\begin{array}{l} \sigma_{+}\left(L\right)=\;\;\sigma_{A}\left(L\right)\;⊖\;\sigma_{B}\left(L\right)=\left(\begin{array}{cc} 0 & 1\\ 0 & 0 \end{array}\right),\\ \sigma_{-}\left(L\right)=\;\sigma_{B}\left(L\right)\;⊖\;\sigma_{A}\left(L\right)=\left(\begin{array}{cc} 0 & -1\\ 0 & 0 \end{array}\right)\;=\;\;\left(\begin{array}{cc} 0 & i\\ 0 & 0 \end{array}\right)\left(\begin{array}{cc} 0 & 0\\ 0 & i \end{array}\right) \end{array}$$ (7)

In both σ_+_(L) and σ_-_(L), the non-reciprocal state *q* is removed (*q*≡0). For the stimulus σ_+_(L), *p* = (0 1) and *q* = (0 0) describe that subjects periodically see their partner’s eyes but not the rest of the face. Based on Eq (1), the functionality of stimulus σ_+_(L) becomes *f*(σ_+_(L)) = *p*. Moreover, the fact that all the truth-values in σ_+_(L) remain real numbers after collation suggests that σ_+_(L) is still a Boolean matrix in logic B1, and is still an exteroceptive stimulus that stimulates the exogenous system.

The result for σ_-_(L) is much more significant and less intuitive. Referring to Appendix A, after being subjected to the collation operation, *p* has truth-value -1 which means “inconsistent” in the three-valued logic. Such inconsistency or “error” in the real Boolean logic B1 can be transformed to another self-consistent imaginary Boolean logic B2 by “-1 = i*i” mapping in algebraic logic. The σ_-_(L) clearly becomes two interoceptive stimulus matrices that contain stimuli π and θ, whose propositions are “I mentalize eyes” and “I mentalize a face without eyes.” Because the first and second imaginary matrices describe π = (0 *i*) and θ = (0 *i*) respectively, the functionality of task σ_-_(L) then becomes *f*(σ_-_(L)) = π∧θ. Thus, the collation operations in Eq (7) and “-1 = i*i” mapping convert the exteroceptive stimuli σ_A_(L) and σ_B_(L) into one exteroceptive stimulus σ_+_(L) and one interoceptive stimulus σ_-_(L). Most significantly, the σ_-_(L) becomes the cause for emergence of the endogenous response due to inconsistency in the exogenous response.

As a side note, the collations of the stimulus matrices for the right-side subject group yield different composite stimuli than those from the left-side subject group in Eq (7) due to the phase difference in the stimulus time courses in Fig 1c:

$$\begin{array}{l} \sigma_{+}\left(R\right)=\;\sigma_{A}\left(R\right)\;⊖\;\sigma_{B}\left(R\right)=\left(\begin{array}{cc} 0 & 1\\ -1 & 1 \end{array}\right)\;=\;\left(\begin{array}{cc} 0 & 1\\ 0 & 1 \end{array}\right)+\left(\begin{array}{cc} 0 & 0\\ i & 0 \end{array}\right)\left(\begin{array}{cc} i & 0\\ 0 & 0 \end{array}\right),\\ \sigma_{-}\left(R\right)=\;\sigma_{B}\left(R\right)\;⊖\;\sigma_{A}\left(R\right)=\left(\begin{array}{cc} 0 & -1\\ 1 & -1 \end{array}\right)\;=\;\left(\begin{array}{cc} 0 & 0\\ 1 & 0 \end{array}\right)\;+\;\left(\begin{array}{cc} 0 & i\\ 0 & i \end{array}\right)\;\left(\begin{array}{cc} 0 & i\\ 0 & i \end{array}\right) \end{array}$$ (8)

Unlike the left-side composite stimuli, σ_+_(R) is not a pure exteroceptive stimulus, and σ_-_(R) is not a pure interoceptive stimulus. Therefore their responses are mixtures of the exogenous and endogenous systems. This is because the initial phase in a complex number time series bears significant information. Meanwhile, since time-invariant feature in a dual system could be a more complicated issue beyond the scope of this study, no time shift in σ_B_(R) to match the σ_A_(R)’s phase is performed here. Fortunately, the BOLD responses that are statistically derived from the left-side group by mixed-effects analysis should apply to the right-side subject group in a standard brain space. Thus, in all the later text the σ_+_ and σ_-_ represent only σ_+_(L) and σ_-_(L) in this study.

### The responses to the composite stimuli

The logical deductions of the stimulus-response transformations (σ-Ω) for the composite stimuli (σ_+_ and σ_-_) are listed in Table 1 as A-B and B-A, and their derivations are detailed in Appendix C. Based on the transformations, the cerebral responses for the composite stimuli are expressed in truth-table in Table 2 as Ω_+_ = *x*∧¬*y* and Ω_-_ = ¬(ξ∧ψ)∧¬ψ. Notice that Ω_+_ is only composed of the exogenous states, while Ω_-_ is only composed of the endogenous states. Thus, the dual logic deduction results explicitly formulates that the exteroceptive stimulus σ_+_ causes the exogenous response Ω_+_ and the interoceptive stimulus σ_-_ causes the endogenous responses Ω_-_.

According to Table 2, the exogenous system activation (Ω_+_ = 1) is the result of a reciprocal state (*x* = 1, *y* = 0), where the non-reciprocal *y*-state is suppressed. However, the endogenous system activation (Ω_-_ = *i*) is a superposition of both the reciprocal state (ξ = *i*, ψ = 0) and a default state (ξ = 0, ψ = 0), albeit the non-reciprocal ψ-state suppression. Note that the default state is neither reciprocal nor non-reciprocal. It is an intrinsic system embedded in the endogenous system, activated when the endogenous system emerges during dyad’s reciprocal interaction. With logical rigor and determinism, the Ω_+_ and Ω_-_ mark the cerebral regions where reciprocity occurs during eye contact, which can subserve a cerebral coordinate for reciprocal interaction (CCRI).

## Methods

### Participants

The Princeton University institutional review board specially approved this study (IRB #4946). All participants gave informed and written consent based on the approved IRB. A total of 19 pairs (38 individuals) of subjects were enrolled in the dfMRI experiment. Most of the participants were university students. Their average, standard deviation, maximum, and minimum age were 22, 5, 33, and 18 years old. The numbers of pairs for female-female, male-female, male-male were 11, 4, and 4. There were 12 females and 7 males on the left side, and 14 females and 5 males on the right side. Prior to the scans, each participant took a behavioral test called “Inclusion of Other in the Self Scale” (IOS) [19], for evaluating the closeness between the partners. The average scores for the left and right side subjects were 4.89 and 4.95 with standard deviations 1.45 and 1.39, which indicates the balanced intimacy level between left and right side subject groups. The IOS scale was 1 to 7 (7 for being the closest).

### Experimental procedures

All subjects were instructed to be natural and calm as much as possible while maintaining spontaneous facial expression during scanning. In task A, when they heard the verbal instruction “close”, the dyad should close their eyes simultaneously; when they heard the instruction “open”, the dyad should open their eyes simultaneously and make eye contact with either a direct gaze or an averted gaze according to their comfort. In task B, when they heard the instruction “one”, the right-side subject should switch to eyes open and the left-side subject should switch to eyes closed. When they heard the instruction “two”, the right-side subject should switch to eyes closed and the left-side subject should switch to eyes opened. All verbal instructions were delivered through headphones.

### Data acquisition

All functional, anatomical, and field mapping images were acquired on a 3T Siemens Magnetom Skyra MRI scanner (Siemens, Erlangen, Germany) using a custom-made dual-head coil [1]. The functional protocol was a gradient-echo EPI. Its spatial parameters were voxel size ~4mm×4mm×4mm, FOV 500mm×254mm, slice thickness 4mm, 32 transverse slices, and slice order interleaved. Its temporal parameters were TR 2000ms, TE 30ms, echo spacing 0.52ms, 200 repetition volumes. Its 4D sampling matrix was 128×64×32×200. The field mapping protocol was a double echo gradient-echo sequence with TE1 4.92ms, TE2 7.38ms, TR 1230ms, echo spacing 0.58ms, flip angle 60, and the spatial sampling region identical to the EPI. The anatomical protocol was a 3D MPRAGE with voxel size 2mm×2mm×2mm, 96 coronal slices per slab, FOV 500mmx250mm, and 3D sampling matrix 256x128x96. In every dfMRI experiment, in addition to the task A and B described in Fig 1c, a functional baseline data was also collected, in which the dyad were closing their eyes during entire scan session.

The factory-specified homogeneous static magnetic field region for the Skyra is an ellipsoid with three axes: 50cmx50cmx45cm. When two medium-sized subjects are positioned as in Fig 1a, both of their brains are just able to fit in the ellipsoid, so that dyadic anatomical images can fully capture both brains as shown in Fig 1b. However, because the EPI sequence is more sensitive to field inhomogeneity, the functional images often miss part of the occipital lobe, see Supporting Information (S1 File). Given that the main focus of this study is to identify social brain, such as empathy or mentalization networks, excluding the occipital lobe here bears minimal consequences for now.

### Data post-processing for the CCRI

The BOLD responses for the task A and B (Ω_A_ and Ω_B_) were calculated by group analysis of general-linear-model (GLM) regression. The exogenous and endogenous systems (Ω_+_ and Ω_-_) were estimated by paired t-test comparison. Both were implemented by the software package FSL (Oxford University, UK) [20]. The CCRI was the binary masks of the Ω_+_ and Ω_-_.

In the preprocessing, each of the dyadic 4D functional data sets from the task A and B was first split into two monadic data sets for separating the left and right subjects. Then all of the monadic 4D data sets were put through motion correction, slice time correction, and brain extraction, as well as spatial smoothing with HMFW 8mm and temporal high pass filtering with a cut-off period of 60s. Each of the dyadic 3D anatomical and field mapping data sets was also split to two monadic data sets for separating the left and right subjects. Then all monadic 3D data sets were put through bias field correction and brain extraction. Note that during each data split, the sampling volume and coordinate information in the header of each monadic data file were reset in order to properly register to the standard MNI152 [21] template. Meanwhile, since the brain orientations in dyadic data are different from the orientation of the MNI152 template, to avoid rotating all the functional data sets to adapt to the MNI152 template, the MNI152 template was rotated -90° for the left subject registration and 90° for the right subjects registration. The inversed rotation matrices and the new center offset were included in the file header so that the rotated standard images retained an accurate atlas label reading from the Harvard-Oxford Atlas [22]. The registration contained three steps: First, the “weighted registration” function in FSL was used to initially register the functional data to the bias-corrected magnitude images in field mapping in six degrees of freedom (DOF), with the mask that 25% of the posterior part of image was masked out to avoid signal drops in the occipital lobes and to maintain that the frontal, temporal, and parietal lobes were accurately registered to the MNI152 standard brain. Second, the initially registered images were then registered to high-resolution anatomical images with 12 DOF. Third, the high-resolution images were registered to the standard MNI152 template with 12 DOF.

**Fig 3:**
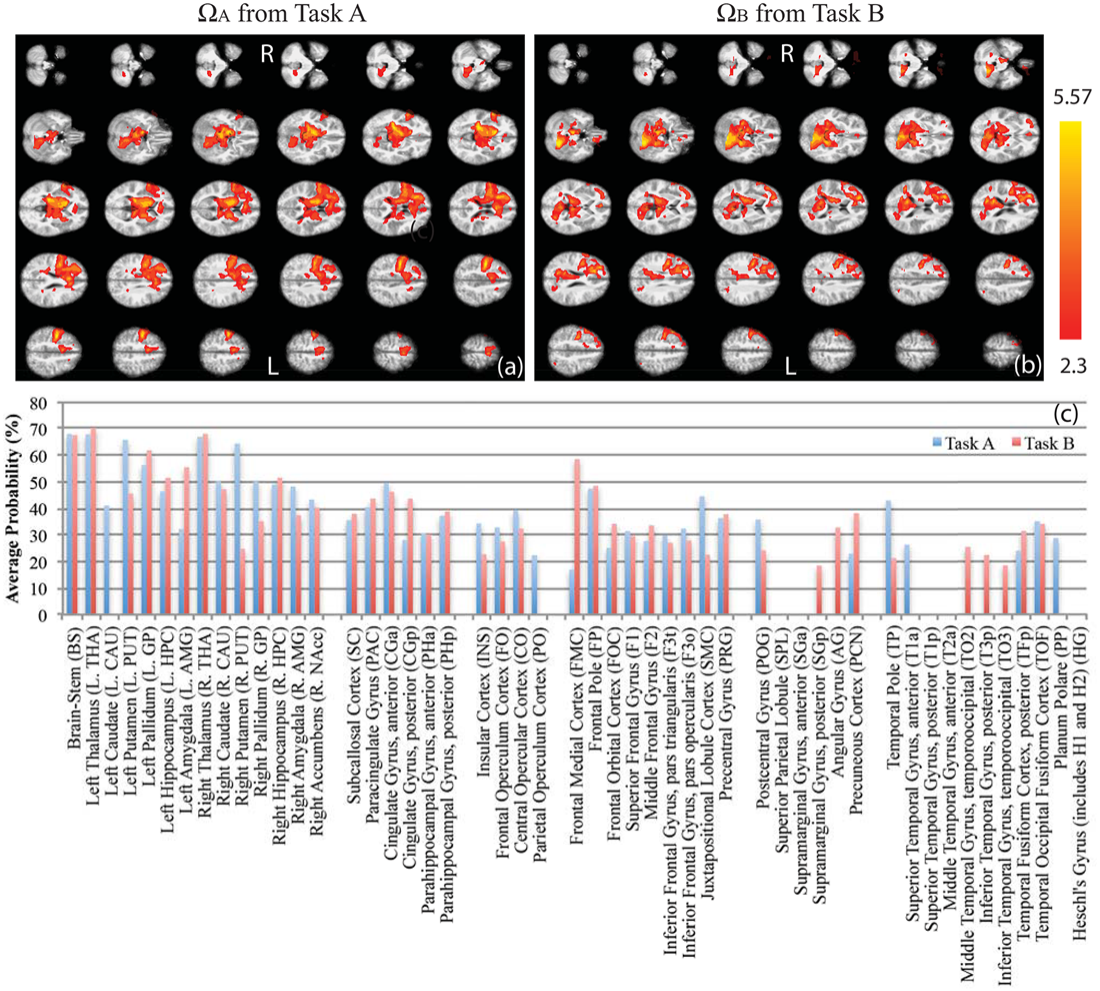
The group average GLM results for all left-side subjects. The (a) is the BOLD response in the task A, Ω_A_; the (b) is the BOLD response in the task B, Ω_B_; the (c) is the probabilistic atlas label distributions for the activations in both the task A and the task B. Note that the abbreviations of the labels’ names in this study are defined here.

**Fig 4:**
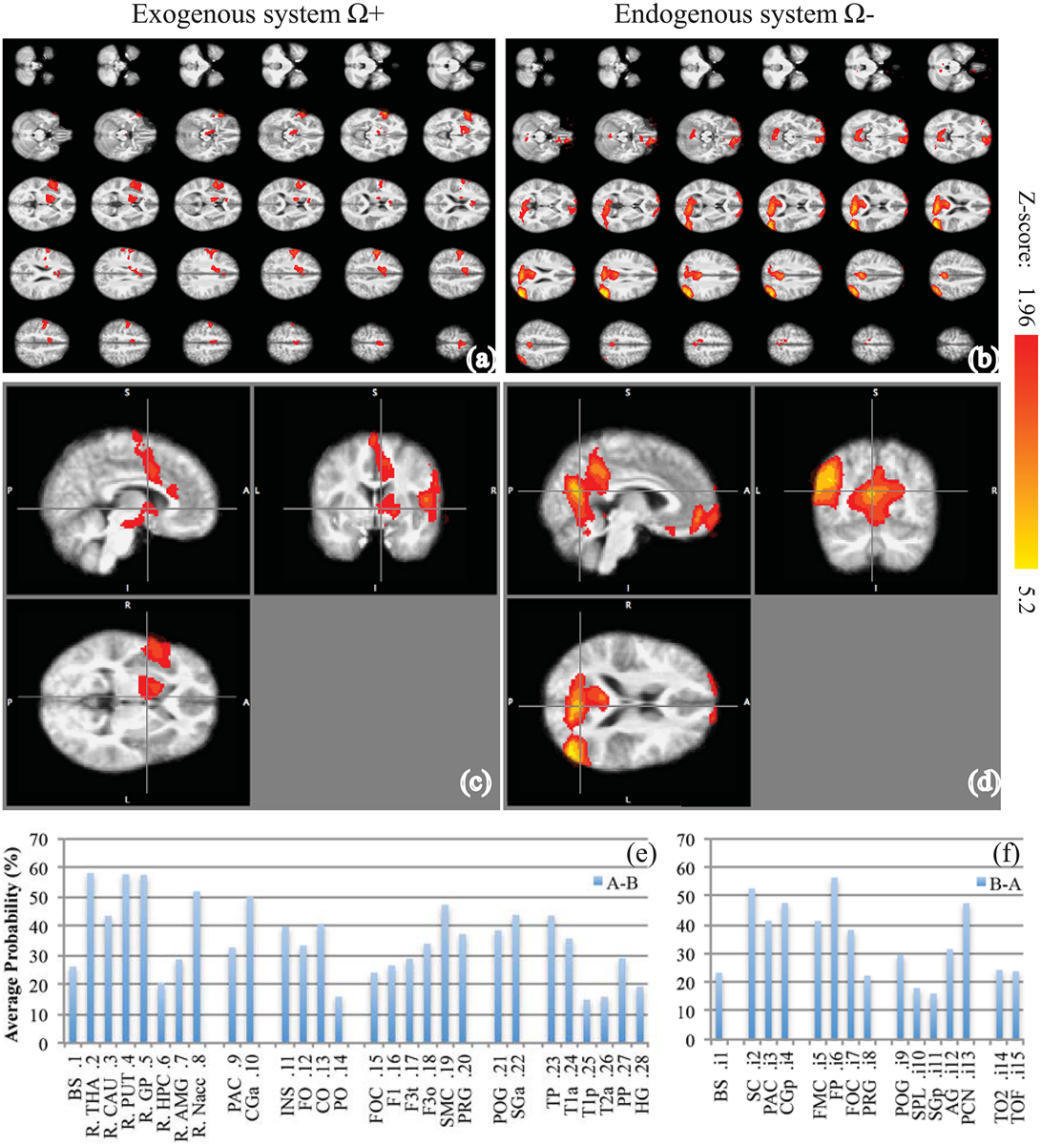
The reciprocal BOLD responses due to eye contact. The (a) is the exogenous responses in which Ω_+_ = 1 when (*x* = 1, *y* = 0); the (b) is the endogenous responses in which Ω_-_ = 1 when (ξ = 1, ψ = 0) and (ξ = 0, ψ = 0). Here the (c) and (d) are the three orthogonal sections of the (a) and (b) respectively; the (e) and (f) are the probabilistic atlas label distributions for the exogenous-endogenous dual system, where the exogenous labels are indexed by real number, and the endogenous labels are indexed by imaginary number.

After data preprocessing, Group GLM analysis was performed on the preprocessed monadic data sets from both task A and B. The hemodynamic response functions (HRF) were the block waveforms in Fig 1c convolved by the first three eigen-components in the linear optimal basis set [23]. The group average GLM for Ω_A_ and Ω_B_ were shown in Fig 3a and 3b. The paired t-test comparison for Ω_+_ and Ω_-_ were shown in Fig 4a–4d. The activation labeling was based on the “Harvard-Oxford cortical structural atlas” and the “Harvard-Oxford subcortical structural atlas,” which are built-in features of FSL. In the end, the masks of Ω_+_ and Ω_-_ (1 for activation, 0 for no activation) became the CCRI. The atlas labels of Ω_+_ and Ω_-_ became the coordinate ticks in the CCRI, where exogenous and endogenous labels are indexed by real and imaginary numbers respectively, as shown in Fig 4e and 4f.

### An example for applying the CCRI

During eye contact, brain synchronization induced by reciprocal interaction can be decomposed into multiple coupling modes. Each mode represents a different interactive mechanism between dyadic brains. One way to estimate such coupling modes is to apply the independent component analysis (ICA) to the dyadic data from the task A. (Because only task A has eye contact.) The results of ICA are a set of independent components (IC) in which both reciprocal and non-reciprocal responses are entwined. By projecting the IC onto the CCRI, the non-reciprocal responses should be filtered out, and the reciprocal responses in each IC remain.

**Fig 5:**
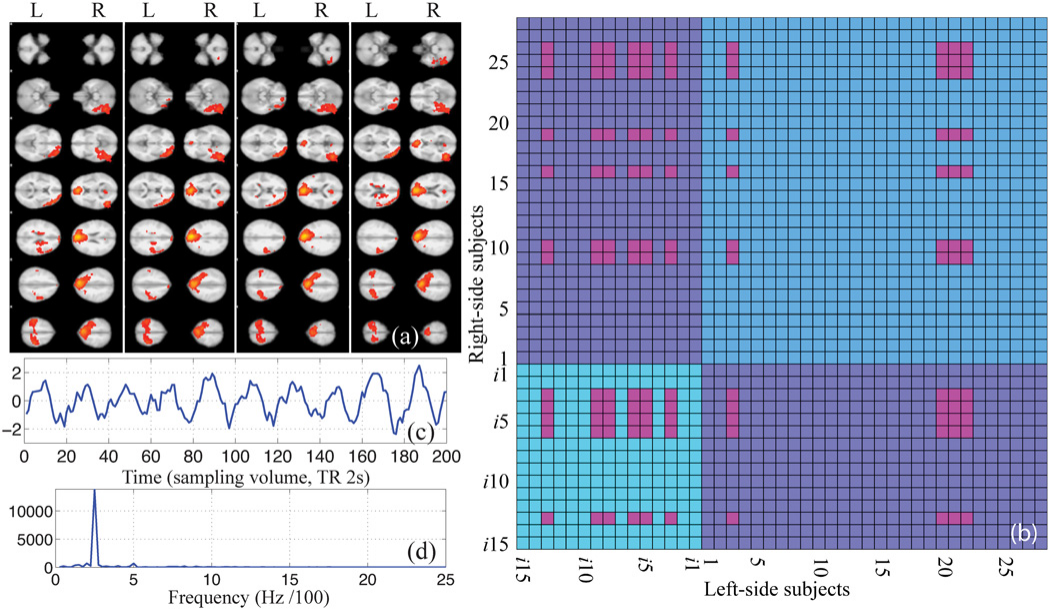
One of the dyadic brain-to-brain coupling modes. The (a) is one of the independent components derived from the 19 data sets in the task A by group-level ICA. The (b) is the 2D matrix representation of the coupling mode after the IC is projected onto the CCRI. Note that each axis has real and imaginary regions that correspond the exogenous and endogenous labels respectively. All complex numbers and their corresponding labels are listed in the Fig 4e and 4f. Here the vertical axis is for right subjects and the horizontal axis is for left subjects. The (c) and (d) are the temporal course and the frequency response of the synchronized process that represented by this IC.

The 19 dyadic data sets from the task A were processed in following three steps: First, since FSL can only handle monadic data, in order to assign labels to a dyadic IC with FSL, the dyadic data was first split and preprocessed, then the left and right monadic data were registered to the rotated left and right MNI152 standard templates respectively by using the same procedure as in the group GLM, then merged to form a registered dyadic data set. Second, group level tensor-ICA for all 19 registered dyadic data sets was computed by FSL/melodic and yielded 35 ICs. Fig 5a selectively displays one of the 35 ICs. Third, in order to project the ICs onto the CCRI, each dyadic IC was split into two monadic data sets again. The split ICs for the left and right subjects were separately multiplied by the properly oriented CCRI first, and then processed for atlas labeling. The activated labels for the right subjects were indexed as a vertical axis. The activated labels for the left subjects were indexed as a horizontal axis. In this way, each coupling mode can be quantified by a matrix based on the labels in the CCRI, as shown in Fig 5b.

## Results

First of all, applying GLM on the baseline data (dyads closed eyes in entire scan) with the regressors in Fig 1c, no BOLD activation was observed in dyads, which suggests that neither non-visual stimuli nor physiological coupling contribute to the BOLD responses in the task A and B.

### The exogenous and endogenous systems Ω_+_ and Ω

The BOLD responses for the original tasks A and B, Ω_A_ and Ω_B_ respectively, are the group averages of GLM with the data from all the left subjects, as shown in Fig 3a and 3b, where the inference threshold is Z>2.3 and p-value<0.05. Note that no voxels exhibiting negative BOLD responses were observed in the Ω_A_ and Ω_B_. (Here the right-side subject group analysis is ignored because the logical deduction in Eq (8) suggests that its paired t-test comparisons between Ω_A_ and Ω_B_ may not yield pure exogenous or pure endogenous response due to their regressors’ phase.) The probabilistic atlas label [22] distributions for the activated brain regions in the MNI152 standard template [21] are listed in Fig 3c, in which the probability of each label is the average probability over the conjunction of the label’s mask (probability>15%) and activation maps (Z>2.3). The selected labels are grouped in six regions: the subcortex, the limbic lobe, the insula/operculum, the frontal lobe, the parietal lobe, and the temporal lobe. All abbreviations of the names of the labels are in Fig 3c. Due to field inhomogeneity artifacts in the partial occipital lobe, all occipital labels are removed in this list.

The BOLD responses of the exogenous and endogenous system for reciprocal interaction in eye contact, Ω_+_ and Ω_-_, were estimated by a paired t-test comparison between Ω_A_ and Ω_B_ in a group analysis. Both the exogenous system Ω_+_ and the endogenous system Ω_-_ are shown in Fig 4a and 4b, and their cross-section views in Fig 4c and 4d. Although the t-test threshold for generating Ω_+_ and Ω_-_ is lowered to Z>1.96 and p-value<0.05 for scoping finer differences, the inference threshold remains Z>2.3 and p-value<0.05 in clustering Ω_+_ and Ω_-_ to identify the dual system. The probabilistic atlas label distributions for Ω_+_ and Ω_-_ in the MNI152 standard template are listed in Fig 4e and 4f respectively, where the probability of each label is the average probability over the conjunction of the label’s mask (probability>15%) and the activation maps. Note that, to distinguish the dual systems, the labels in Fig 4e are indexed by real numbers, and the labels in Fig 4f are indexed by imaginary numbers. In a cluster analysis, three clusters are identified in Ω_+_; and seven clusters are identified in Ω_-_. The names, vicinities, voxel sizes, maximal Z-scores, and MNI152 coordinates of the clusters are listed in Table 3. Here, cluster size > 64 voxel, given that the spatial smoothing filter is 8×8.

**Table 3:** The organization of the exogenous-endogenous system.

Cluster	Average probability>15%	Voxels	Max	MNI152	MNI152
number	Z>2.3	(#>64)	Z	max Z	center of gravity
Exogenous System Ω+:					
1	INS, FO, CO, PO	2065	4.47	(62, 16, -10)	(55.1, 5.43, 10.7)
	FOC, F3t, F3o, PRG				
	POG, SGa				
	TP, T1a, T1p, T2a, PP, HG				
2	BS,THA,CAU,PUT,GP,HPC,AMG,Nacc	901	3.32	(8, 8, 38)	(12.5, 3.26, 19.5)
	PAC, CGa				
	F1, SMC, PRG				
3	F1, SMC, PRG	326	2.96	(0, -2, 70)	(-2.55, -5.93, 71.7)
Endogenous System Ω-:					
1	CGp, PRG, POG,PCN, TOF	5125	4.39	(-4, -72, 22)	(0.603, -62, 18.5)
2	SPL, SGp, AG, TO2	3081	5.2	(-46, -70, 32)	(-46, -71.2, 27.2)
3	PAC, CGa; FMC, FP, FOC	1166	3.7	(-6, 52, -8)	(-10.8, 57.7, -8.42)
4	FP	428	3.51	(16, 68, 8)	(20, 65.1, 17.2)
5	FP	189	3.17	(22, 66, -12)	(25.6, 64.7, -14.7)
6	SC, FMC, FP, FOC	117	3.25	(-12, 18, -30)	(-9.74, 22.4, -26.3)
7	FP	89	3.15	(46, 50, -18)	(44.7, 50.5, -16.1)

Elucidated by the logical deductions in Table 2, here Ω_+_ is the data-driven exogenous system that responds to reciprocal exteroceptive stimulus *p*; and Ω_-_ is the data-driven endogenous system that are the responses to both reciprocal interoceptive stimulus π and emergence of the default state during eye contact. The binary masks of the Ω_+_ and Ω_-_ define the data-driven CCRI.

### An example of using the CCRI to compute dyadic coupling modes

The probabilistic ICA analysis (FSL/melodic) was applied on the dyadic (not split) data sets from the task A. With the mixture-modeling threshold set to 0.8, the group level ICA for all 19 paired data sets yielded a total of 35 ICs. 22 of the ICs had temporal courses that corresponded to the regressor of task A in Fig 1c and had resonance peaks at 0.025Hz in their frequency response, as in Fig 5c and 5d, which indicated that these ICs were in synchrony with eyes opening and closing. Of the 22 eye-contact-related ICs, 14 of them had single and robust resonance peaks, while the other eight had multiple frequency modulations either due to motions or related to ventricles. Of the 14 robust eye-contact-related ICs, four of them were monadic (only activated on one side of subjects), and ten of them were dyadic. These ten dyadic ICs were selected as the exploratory coupling modes. Projecting these ten ICs onto the CCRI resulted in the coupling modes that are in dual system forms and likely contain only the reciprocal responses and default state activations. Here, only one of the ten coupling modes was chosen to demonstrate the application of the CCRI. The complete dyadic coupling mode analysis will be the subject of on-going research due to its extensive contents.

IC #29 was chosen to briefly exemplify the application of the CCRI. Fig 5a is the original IC #29. After both the left- and the right-side subjects’ activation maps were projected onto the CCRI, the left-subjects’ labels were distributed on both the endogenous (PCN/PAC, FMC/FP, PRG/POG) and the exogenous (CAU, PRG/POG, SGa). The right-subjects’ labels were distributed on both the endogenous system (PCN/PAC/CGp, FMC/FP) and the exogenous system (CGa/PAC, SMC/F1, T1a/T1p/T2a), as shown in Fig 5b. This mode seems to illustrate the medial frontoparietal activation in social cognition articulated in the Ref. (8), in which the endogenous (FMC—PCN) between the left-subjects and the right-subjects are synchronized. In addition, Fig 5b seems to also suggest that this endogenous coupling may be mediated by their exogenous coupling between the left-subjects and right-subjects. The temporal course of this process and its prominent 0.025Hz peak in its frequency response in Fig 5c and 5d indicate that this brain-to-brain synchronization occurs when the pairs have eye contact.

## Discussion

### The dual logic for the dual systems

By expanding Boolean logic, the dual logic can symbolically formulate dual systems with an emergence mechanism. It introduces two original fundamental concepts: First, although it has been elaborated in literature that dual processes operate in significantly different ways in social cognition [24,25], such differences have not been rigorously formulated at the level of formal logic. Here, given the dual system model in Fig 2, as well as the binary nature of tasks (eyes open/closed) and responses (ON/OFF) in the experiments in Fig 1, the logical connectives between stimuli and responses in the exogenous and endogenous dual systems can be explicitly defined: The exogenous process operates with material equivalence (↔); the endogenous process operates with exclusive disjunction (⊕). Based on this definition, the exogenous process behaves as that of “if and only if a stimulus occurs, then a response is activated”; the endogenous process behaves as that of “if and only if a stimulus is unexpected, then a response is activated”. These are the precise characterizations for the “thermostat” aspect of the relation between the reflexive and reflective systems [26]. The formal logic description of this relation is the duality principle in which ↔ and (⊕) are a dual pair that are bonded by De Morgan’s law. In the dual logic model for the experiment in Fig 1, the “↔” operation in the exogenous system is manifested in the first and second axioms; the “⊕” operation in the endogenous system is manifested in the third axiom. The first and second axioms are self-evident, which are detailed in Appendix B. The third axiom is a conjecture based on both a heuristic “thermostat” description of the dual system and the duality principle. It also seems to be consistent with recent evidence that suggests that ⊕ might be a slower and rule-based way of human brain function [17,18].

The second original concept is the use of complex binary numbers as logical truth-values for the dual system: (1, 0) for the exogenous system and (*i*, 0) for the endogenous system. At the fundamental algebraic logic level, the complex truth-value enables formulating emergence in dual systems. As detailed in Appendix A, the logic B1 and B2 are two closed binary logic sets for dual systems without concerning the transition process details between dual systems. However, if one has to logically formulate such a transition between the exogenous and endogenous system, binary logic may become inadequate. First, to formulate a basic process of comparison between expectation and proprioception, a three-value logic operation, collation, is defined in Appendix A. Its three truth-values are 1, 0, and -1 for true, false, and inconsistent. Comparing a proprioception to expectation in the exogenous system, if the result of collation is either 1 or 0, then the impact of the proprioception remains in the exogenous system; if collation yields -1, the inconsistency in the exogenous system triggers emergence of the endogenous response. Second, to avoid the degeneracy in describing that brain regions can be shared by the dual systems, the logical truth-value in the endogenous system should be orthogonal to its peer in the exogenous system. In algebraic logic, this can be achieved by a simple mapping -1 = i*i in a five-value logic, see appendix A. Thus, emergency can be formulated in two steps: collation and -1 = i*i mapping.

In this specific experimental situation in which dyads are locked in eye contact and isolated from other mutual or environmental stimuli, given that the tasks (eye open/close) and responses (ON/OFF) are binary, the three axioms in Eqs (2–4) establish the foundation for dual logic deduction. Based on the axioms, the exogenous and endogenous systems can be identified, the non-reciprocal responses can be suppressed, and the existence and emergence of the default state in the endogenous system can be predicted, all by deductive approach.

### The dual systems per the dual logic

Conceptually, the exogenous-endogenous dual systems can be distinguished by their stimuli being either exteroceptive or interoceptive. Logically, the dual systems operate with different logic connectives that are either material equivalence or exclusive disjunction. In any case, they may slightly deviate from the traditional automatic-controlled dual system in social psychology [26–29]. Their functionality resembles that of the reflexive-reflective dual systems [26]. Their organization is close to the data-driven externally-focused and the internally-focused dual system framed in cognitive neuroscience [8].

The dual system responses Ω_+_ and Ω_-_ in Fig 4 and Table 3 not only are consistent with the original neural correlates of the externally-focused and internally-focused dual processes [8], but they also provide more complete brain organizations for the dual system. For the exogenous system where Ω_+_ = 1 if and only if (*x* = 1, *y* = 0), not only does Ω_+_ confirm lateral frontoparietal activation [8], but it also identifies the regions that largely overlap with the mirror neuron system (F3o, SGa) [30], imitation circuitry (T1p, mirror neuron) [31], and the social empathy network (INS, CGa, imitation circuitry) [32], as well as some afferent and efferent subcortex and motor cortices. For the endogenous system where Ω_-_ = 1 if (ξ = 1, ψ = 0) or (ξ = 0, ψ = 0), not only does Ω_-_ confirm the medial frontoparietal activation (FMC and CGp/PCN) [8], but it also adds the left AG and FOC to the mix. This activation pattern resembles the DMN [33], except for its left hemisphere dominant lateral asymmetry. Given that the DMN is usually in resting-state and its function seems to be self-referential (ξ = 0) [34], its activation may offer evidence for the superposition of the reciprocal social state (ξ = 1, ψ = 0) and the default state (ξ = 0, ψ = 0), which is predicted in Table 2.

Such dual systems seem to be only activated in face-to-face reciprocal eye-contact. In a separate experiment, described in the Supporting Information (S2 File), gazing to the eyes in a pre-recorded face video did not prompt the same dual system activations, most likely due to lack of reciprocity. In that case, there is no lateral frontoparietal activation, especially no insular activation, in the exogenous system (A-B); and no medial frontoparietal activation in the endogenous system (B-A)—the DMN remains in resting-state. So it is fair to say that dfMRI can reveal some social brain behaviors that other methods cannot. The fundamental difference between the dfMRI and other methods is that it can capture the unfiltered reciprocity.

### Emergence mechanism

Although there could be many pathways between the exogenous and endogenous systems, the most obvious transition between the dual systems seems to happen at the cingulate. According to some influential theories, the anterior cingulate (CGa) may be engaged in monitoring conflicts with expectations [35]; the posterior cingulate (CGp) may be engaged in regulating the balance between internally and externally directed cognition [36] and in retrieving autobiographical memories [34]; the cingulate and paracingulate may be responsible for agent recognition in the social domain (“me” and “not me”) [37]. From an emergence point of view, these previous observations and theories could be nicely explained by the dual system and dual logic. Based on the data-driven CCRI, as shown in Fig 4, CGa and PAC are in the exogenous system while CGp and PAC are in the endogenous system. The logical description of the emergence from the exogenous to endogenous system has two steps: the collation that compares proprioception with expectation, and the -1 = i*i mapping that transcends exogenous inconsistency to the endogenous response. Apparently the collation operation seems to be the logical expression of monitoring conflict, so it should occur in CGa. From the truth table of the collation shown in Appendix A, if the proprioception is the same as the expectations, then the collation results are false (0), which indicates that no conflict is detected and no action is needed. If there is no expectation but proprioception is positive, then the collation result is true (1), the truth-value remains a real number, which suggests an exogenous activation. Most interestingly, if there is an expectation but no proprioception, then collation yields inconsistency (-1), which means conflict or error. Such inconsistency in the exogenous system prompts emergence of the endogenous system by the -1 = i*i mapping. Given the function of the CGp in regulating the balance between exogenous and endogenous, this mapping likely occurs in the CGp. Overall, during eye contact, saccade of the partner presents constant unexpected proprioception, which results in continuous inconsistency from collation and “-1 = i*i” mapping. Such dynamic monitoring conflict and balancing the dual systems constantly recruit the CGa and CGp, and make the cingulate an agent-specific emergence site.

## Conclusions

The dual logic is proposed for explicitly formulating the dual systems and emergence mechanism between the dual systems. It’s one of the few initial attempts to use the closed logic system to analyze agent-specific observations, especially when the observer is also being observed. It offers a deterministic approach to complement the existing common statistical approaches in neuroimaging analysis. By applying the dual logic in the dfMRI experiment design and analysis, the data-driven exogenous and endogenous systems that delineate the dual logic deduction provide a generic CCRI in which the exogenous and endogenous system consist of mainly the empathy network and mentalization network respectively. Moreover, the logical interpretation of the data-driven endogenous activations elucidates the intrinsic and social characteristics of the DMN; the logical formulation of the transition between the exogenous and endogenous system elicit the role of CG in agent recognition in the social domain. Overall, the dual logic deductions are supported by the dfMRI experimental results and are consistent with current literature. Both the theoretical framework and experimental method set a stage to formally apply the scientific method in studying complex social interaction.

## Appendices

### A. Construct of a dual system with abstract algebraic logic

In the well-established abstract algebraic logic approach [38], a logic problem can be transformed to algebraic forms, and resolved with algebra, and transformed back to a logic solution. Given the dual system model in Fig 2, as well as the binary tasks (eyes open/closed) and responses (ON/OFF), the binary Boolean logic is mostly sufficient to formulate the dfMRI experiment in this study. Here the definition of the original Boolean logic is given as:

B1 =  〈w f f; 0,  1;  ⊕, ∧, ¬〉. (A1)

Here *wff* means well-formed formula. The truth-values are 1 for true and 0 for false. Although a two-value logic can have a total of 2^4^ logic operations, all of them can be composed by a minimum set of operations ⊕, ∧, and ¬. The B1 can be transformed to the Boolean algebra

$$Br\;=\;Alg\left(B1\right)\;=\;\;\left\{F\left(x\right);x\in \;\;{\left[0,\;1\right]}_{b};\;+,\;*,\;\;1+x\right\}.$$ (A2)

**Fig 6:**
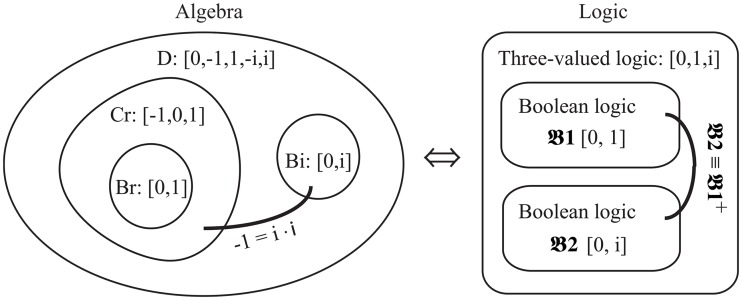
The transformation between the algebra and the logic.

Here, the **Alg** is the transformation from logic to algebra. The *F*(*x*) is the algebraic expression over variables *x*, and *x* has binary values 0 or 1. The logic operations ⊕, ∧, and ¬ coincide with the arithmetic operation +, *, and 1+x, meaning they have the same truth-table operation respectively. Note that addition (+) is performed modulo 2 here. As shown in Fig 6, the Br is a subset of a three-valued algebra

$$Cr=\;\;\left\{F\left(x\right);x\in \;{\left[1,\;\;0,\;\;-1\right]}_{t};\;\;+,\;\;-,\;*,\;\;1\;+\;x\right\}$$ (A3)

Here *x* has ternary values 1, 0, and -1. The arithmetic operation subtraction (-) is also performed modulo 2. Its corresponding logic operation (with the same truth-table operation, Table 4) is defined as collation with symbol ⊖. The logical meaning of 1 is true, 0 is false, and -1 is inconsistent. The practical explanation of the collation (*β* ⊖ *α*) can be described as α being the expectation value, β being the proprioception value. If the proprioception matches the expectations (either α = β = 0 or α = β = 1), then no action is needed (*β* ⊖ *α* = 0). However, if the proprioception comes as unexpected (α = 0, β = 1), then the proprioception will prompt action to address the unexpected (*β* ⊖ *α* = 1). More interestingly, if the expectation is there but the proprioception is not (α = 1, β = 0), then no proprioception can prompt action to address the unexpected, which results in inconsistency or “error” (*β* ⊖ *α* = −1).

**Table 4:** Collation & Subtraction.

Logic: *β* ⊖ *α*			α	
Algebra: β-α		1	0	-1
	1	0	-1	-1
β	0	1	0	-1
	-1	1	1	0

Meanwhile, Fig 6 also suggests that the algebra Cr is a subset of a five-valued algebra

$$D=\;\left\{F\left(x\right);x\in {\left[1,\;\;-1,\;\;i,\;\;-i,\;\;0\right]}_{q};\;\;+,\;\;-,\;\;\times ,\;\;1\;+\;x{|}_{x\;is\;real},\;\;i+x{|}_{x\;is\;imaginary}\right\}.$$ (A4)

Here *x* becomes a complex number that has quinary values 1, -1, i,-i, and 0. Note that binary algebra has 2^4^ operations, ternary algebra has 3^9^ operations, and quinary algebra has 5^25^ operations.

As illustrated in Table 4, applying subtraction (-) over Br area (the 2-by-2 area at the upper left corner in Table 4) can yield -1. Its corresponding logic explanation is that applying the collation operation will generate an inconsistency in Boolean logic. However, in the algebra D, -1 can be mapped to i*i with the arithmetic multiplication operation. Given the entire algebra D (∀D), there should be existence of a subset algebra Bi (∃Bi),

$$Bi\;=\;\;\left\{F\left(x\right);x\;\in \;{\left[0,\;\;i\right]}_{b};\;+,\;\;⊙,\;\;i+x\right\}.$$ (A5)

Here *i*⊙*i* = i*i ^4^, the imaginary unit self rotates 2π in the complex plane. If the corresponding logic to the algebra Bi is B2, then a subset of B2 that is bonded with B1 by the duality principle can be constructed by

$$B2\;=\;B1^{+}\;=\;\langle w\;f\;f;\;\;0^{+}\;=\;i,\;\;1^{+}\;=\;0,\;\;{⊕}^{+}\;=\;↔,\;\;\wedge^{+}=\vee ,\;\neg \rangle \;=\;\langle w\;f\;f;\;i,\;\;0;\;\;↔,\;\;\vee ,\;\;\neg \rangle .$$ (A6)

Here the superscript + represents dual. The B2 is constructed from the B1 based on the duality principle. Thus, inconsistency in the logic B1 prompts emergency of a consistent logic B2.

To avoid confusion, please note that the reason for using ⊕ to define base operation in B1 is because its logic operation and algebraic operation have the same truth-table. However, when the B1 is used to model the exogenous system for the experiment, its operation is defined as ↔ in the axiom 1 and 2, in which ↔ can be simply expressed as ¬⊕ in the B1. For the similar reason, ↔ is used to define the base operation in B2 because it is the dual of ⊕. When the B2 is used to model the endogenous system for the experiment, its operation is defined as ⊕ in the axiom 3, in which ⊕ can simply expressed as ¬↔ in the B2.

### B. Derivation of the dual logic’s three axioms

To determine whether the logical connectives in the first and second axioms are “material equivalence” (↔), and whether the connectives in the third axiom are “exclusive disjunction” (⊕), the truth-table method is employed to avoid ambiguity of the English language. The complete derivation process is shown in Table 5: First off, assuming that all truth-values in the “connective” columns are unknown, then using the exhaustive method determines their values based on self-evidence and the duality principle. Once the truth-tables are completed, the connectives can be uniquely determined.

**Table 5:** The truth table for the premises.

	Exogenous system			Endogenous system		
Stimulus	task	response	connective	task	response	connective
Seeing eyes	*p*	*x* ∨ *y*	*p* ↔ *x* ∨ *y*	π	ξ∧ψ	π⊕ξ∧ψ
	0	0	1	0	0∧0 = 0	0
	0	0∨1 = 1	0		0∧*i* = 0	
		1∨0 = 1			*i*∧0 = 0	
		1∨1 = 1		0	*i*∧*i* = *i*	*i*
	1	0	0	*i*	0∧0 = 0	*i*
	1	0∨1 = 1	1		0∧*i* = 0	
		1∨0 = 1			*i*∧0 = 0	
		1∨1 = 1		*i*	*i*∧*i* = *i*	0
Seeing face	*q*	*y*	*q* ↔ *y*	θ	ψ	θ ⊕ ψ
without eyes	0	0	1	0	0	0
	0	1	0	0	*i*	*i*
	1	0	0	*i*	0	*i*
	1	1	1	*i*	*i*	0

The first axiom in Eq (2) is basic stimulus-response logic for eye-contact in the exogenous system. Generally, it is not only self-evident but also well articulated that seeing other’s eyes (direct or averted gaze) will prompt either social interaction or emotional responses [10]. In order to use a truth-table to fully describe this event under the experimental condition in Fig 1 within the frame of the dual system model in Fig 2, the “seeing eyes” is expressed as the exteroceptive stimulus (*p*) and its cerebral responses are expressed as reciprocal *x*-state and non-reciprocal *y*-state. Please note that “seeing eyes” (*p*) in the first axiom and “seeing face without eyes” (*q*) in the second axiom are two independent logic variables. They act like two orthogonal axes in describing the task “seeing face”. Thus, when discussing *p* and its responses, none of the responses due to *q* should be any concern. This is important for avoiding confusion in the self-evident explanations.

The connective between *p* and (*x*, *y*) can be derived from the truth-table based on the following self-evidence: In the case that observers cannot see their partners’ eyes, obviously there is neither reciprocal nor non-reciprocal exogenous responses due to exteroceptive stimulus by eye contact. For the proposition that describes this statement, “If *p* = 0, then neither *x* nor *y* can be activated (*x* = 0, *y* = 0)”, its truth-value is true or “1”. For the proposition that contradicts this statement, “If *p* = 0, then there will be activation due to either (*x* = 1, *y* = 0), (*x* = 0, *y* = 1), or (*x* = 1, *y* = 1)”, its truth-value is false or “0”. In the case that the observers can see their partners’ eyes, thus, the exteroceptive stimulus can cause either reciprocal, or non-reciprocal, or both exogenous responses. For the proposition that contradicts this statement “if (*p* = 1), then neither *x* nor *y* can be activated (*x* = 0, *y* = 0)”, its truth-value is “0”. For the proposition that describes this statement, “if (*p* = 1), then there will be activation due to either (*x* = 1, *y* = 0), (*x* = 0, *y* = 1), or (*x* = 1, *y* = 1)”, its truth-value is “1”. A connective with such a truth table is called “material equivalence”, and its formal symbol is ↔.

With the same argument, the connective in the second axiom in Eq (3) can be attributed to “material equivalence” (↔) as well. The third axiom in Eq (4) is basic stimulus-response logic for eye-contact in the endogenous system. Based on the definition in Eq (A6) in Appendix A, the duality principle dictates that all the logic variables and connectives in the endogenous system are simply the dual pairs of the variables and connectives in the exogenous system.

### C. Deduction for the four transformations

The four stimulus-response transformations in Table 1 are the propositional logic descriptions for two original tasks (task A and B) and their two composite tasks (A-B and B-A). Transformation A can be deduced from the first and second axioms:

$$\begin{array}{l} \begin{array}{l} (p↔x\vee y)\wedge \left(q↔y\right)\;\;\text{axiom 1 and axiom 2}\\ p\vee q\;\;\;\;\;\;\;\;\;\;\text{premise}\\ --------------------------- \end{array}\\ (x\vee y)\vee y=x\vee y\;\;\;\text{deduction.} \end{array}$$ (C1)

Here, given the stimulus σ_A_ whose functionality f(σ_A_) = *p*∨*q*, as shown in the Eq (1), the response is inferred as Ω_A_ = *x*∨*y*, which is entwined reciprocal and non-reciprocal states. Transformation B is a trivial case, since it is equivalent to the second axiom. Given stimulus σ_B_ whose functionality f(σ_B_) = *q* based on Eq (1), the response is inferred as Ω_B_ = *y*.

The collation operations in the task space in Eq (7) transform the two exteroceptive tasks σ_A_ and σ_B_ into one exteroceptive task σ_+_ and one interoceptive task σ_-_, where the functionality of σ_+_ becomes *f*(σ_+_) = (*p*∨*q*)¬∧*q*, and the functionality of σ_-_ becomes f(σ_-_) = π∧θ. Note that by definition, the *p* and *q* are dichotomous and independent in stimulus space. Therefore, the *f*(σ_+_) can be logically simplified to “*p*” within the stimulus space, which is also consistent with the σ_+_’s bi-state matrix expression in Eq (7). However, due to the first axiom, the relation between tasks (*p*, *q*) and their responses (*x*, *y*) is not one-to-one mapping, and the operations in the stimulus space and the operations in the response space are not homomorphic. Thus, during the process of deduction from stimulus space to response space, the logical operation steps embedded in the functionality expression of the stimulus (premise) should remain without simplification. The transformation A-B can be deduced from the first and second axiom:

$$\begin{array}{l} \begin{array}{l} (p↔x\vee y)\wedge (q↔y)\;\;\;\;\text{axiom 1 and axiom 2}\\ (p\vee q)\wedge \neg q\;\;\;\;\;\;\text{premise}\\ ----------------------------- \end{array}\\ ((x\vee y\vee y)\wedge \neg y=x\wedge \neg y\;\text{deduction.} \end{array}$$ (C2)

So, given the composite stimulus σ_+_ whose functionality *f*(σ _+_) = *p* (now it can be expressed in its simplified form after deduction), the response is inferred as exogenous Ω_+_ = *x* ∧ ¬ *y*.

The transformation B-A needs to be deduced in two steps: The first is to show that it yields no exogenous response. The second is to establish the emergence of endogenous response. The exogenous part of the transformation B-A is:

$$\begin{array}{l} \begin{array}{l} (p↔x\vee y)\wedge (q↔y)\;\;\text{axiom 1 and axiom 2}\\ q\wedge \neg (p\vee q)\;\;\;\;\;\;\;\;\;\;\;\text{premise}\\ ----------------------------- \end{array}\\ y\wedge \neg ((x\vee y)\vee y)=0\;\text{deduction.} \end{array}$$ (C3)

Logically speaking, the composite stimulus σ_-_ does not yield any exogenous response. As shown in the Eq (7), based on the rule “-1 = i*i” in complex numbers, the transformation B-A formulates an emergence of the interoceptive stimulus σ_-_ whose functionality f(σ_-_) = π∧θ. The endogenous part of transformation B-A can be deduced from the third axiom:

$$\begin{array}{l} \begin{array}{l} (\text{π }⊕\text{ ξ }\wedge \text{ ψ})\;\wedge \;(\text{θ }⊕\text{ ψ})\text{ axiom 3}\\ \text{π }\wedge \text{θ premise}\\ --------------------------- \end{array}\\ \neg (\text{ξ }\wedge \text{ ψ})\;\wedge \;\neg \text{ ψ deduction.} \end{array}$$ (C4)

Given the composite stimulus σ_-_, the endogenous response is inferred to be Ω_-_ = ¬(ξ∧ψ)∧¬ψ. Since the ⊕ logic is somewhat counterintuitive, the detailed deduction of Eq (C4) by truth-table is provided in Table 6 in which ζ = ξ∧ψ. Note that ¬ζ∧¬ψ is the only solution for maintaining conjunction logic.

**Table 6:** Deduction for the transformation in endogenous system.

π	θ	ζ	ψ	π ⊕ ζ	∧	θ ⊕ ψ	π ∧ θ	¬ ζ ∧ ¬ψ
i	i	i	i	0		0		
i	i	i	0	0		i		
i	i	0	i	i		0		
i	i	0	0	i	i	i	i	i
i	0	i	i	0		i		
i	0	i	0	0		0		
i	0	0	i	i	i	i	0	
i	0	0	0	i		0		
0	i	i	i	i		0		
0	i	i	0	i	i	i	0	
0	i	0	i	0		0		
0	i	0	0	0		i		
0	0	i	i	i	i	i	0	
0	0	i	0	i		0		
0	0	0	i	0		i		
0	0	0	0	0		0		

## Supporting Information

S1 Fig A dfMRI raw data set (a) in mosaic format and (b) after FSL preprocessing. (EPS)

S2 Fig The experimental results for the suggested experiment. (EPS)

S1 File A data set of dfMRI acquired with EPI sequence. (DOCX)

S2 File An additional fMRI experiment suggested by one of the reviewers. (DOCX)
