# Correction: Dual Logic and Cerebral Coordinates for Reciprocal Interaction in Eye Contact

**DOI:** 10.1371/journal.pone.0128480

**Published:** 2015-05-11

**Authors:** The PLOS ONE Staff

During typesetting, errors were introduced into the final row of the “Endogenous system” column of Table 1. Please see the corrected Table 1 here.

**Table 1:** The dual logic.

	Exogenous system	Endogenous system
Stimulus States σ	*p* = “I see eyes, direct or averted gaze”	π = “I mentalize eyes”
	*q* = “I see face without eyes”	θ = “I mentalize face without eyes”
Response States Ω	*x* = “exogenous reciprocal social response”	ξ = “endogenous reciprocal social response”
	*y* = “exogenous non-reciprocal affective response”	ψ = “endogenous non-reciprocal affective response”
Axioms	*p*↔*x* ∨ *y*	π⊕ξ ∧ Ψ
	*q* ↔ *y*	θ⊕ψ
Transformations	A: *p* ∨*q* ⊦ *x* ∨*y*	
	B: *q* ⊦ *y*	
	A-B: (*p* ∨*q*) ∧¬*q* ⊦ *x* ∧¬*y*	0
	B-A: *q* ∧¬ (*p* ∨*q*) ⊦ 0	B-A: π∧θ ⊦ ¬ (ξ∧ψ)∧¬ψ

Additionally, there are errors in Eq C4. All uppercase psis (Ψ) should be lowercase (ψ). Please view the corrected equation below.

$$\begin{array}{l} (\pi ⊕\xi \wedge \psi )\wedge (\theta ⊕\psi )\;\;\;\;\text{ axiom 3}\\ \pi \wedge \theta \text{ premise}\\ \text{--------------------------------------------------}\\ \neg (\xi \wedge \psi )\wedge \neg \psi \text{ deduction.} \end{array}$$ (C4)

The publisher apologizes for the errors.
