# Supplementary material for: Dual Logic and Cerebral Coordinates for Reciprocal Interaction in Eye Contact
Source: PLoS One. 2015 Apr 17;10(4):e0121791. doi: 10.1371/journal.pone.0121791 (PMC4401735; doi:10.1371/journal.pone.0121791)
Supplement: S1 File — (DOCX) [file pone.0121791.s003.docx]

**S1 File. A data set of dfMRI acquired with EPI sequence**

Per reviewer request, a raw data set of dfMRI is shown in the Siemens scanner’s mosaic format in S1_Fig. (a). Notice that there are two issues in the data set. First, along the phase encoding direction (vertical direction), there is a slight aliasing due to subjects’ uncontrollable motion for the given posture. Two steps were taken to successfully minimize such motion artifact: (1) The FOV along vertical direction was set large enough so that the aliasing was outside of brain region. (2) FSL’s brain extraction and motion correction tools can significantly remove the impact of such motion artifact, as shown in S1_Fig. (b). Second, severe signal reduction occurred around the part of occipital lobe due to the magnetic field inhomogeneity and the gradient non-linearity in these regions. No occipital activity was included in this study. A new generation of hardware solution for this issue is under investigation.

**S1_Fig. A dfMRI raw data set** (a) in mosaic format and (b) after FSL preprocessing.
