# Supplementary material for: Dual Logic and Cerebral Coordinates for Reciprocal Interaction in Eye Contact
Source: PLoS One. 2015 Apr 17;10(4):e0121791. doi: 10.1371/journal.pone.0121791 (PMC4401735; doi:10.1371/journal.pone.0121791)
Supplement: S2 File — (DOCX) [file pone.0121791.s004.docx]

**S2 File. An additional fMRI experiment suggested by reviewer**

To prove that the brain responses to reciprocal eye contact is fundamentally different from the brain responses due to non-reciprocal eye gazing, a conventional fMRI experiment was performed by using pre-recorded video as stimulus. Nine pairs of subjects participated this experiment. The subject-under-test and the person-in-video performed the task A and B in Fig. 1c. Using the same GLM group analysis described in the methods section, the results of A-B and B-A are shown in S2_Fig. Notice that the results of A-B and B-A in this experiment are significantly different from the ones in dfMRI in Fig. 4. There is no lateral frontoparietal activation, especially no insular activation, in the exogenous system (A-B), and no medial frontoparietal activation in the endogenous system (B-A) – the DMN remains in resting-state. So it is fair to say that dfMRI can reveal some social brain behaviors that other methods cannot. The fundamental difference between the dfMRI and other methods is that it can capture the unfiltered reciprocity.

**S2_Fig. The experimental results for the suggested experiment**
